# Photobiomodulation Promotes Hippocampal CA1 NSC Differentiation Toward Neurons and Facilitates Cognitive Function Recovery Involving NLRP3 Inflammasome Mitigation Following Global Cerebral Ischemia

**DOI:** 10.3389/fncel.2021.731855

**Published:** 2021-08-20

**Authors:** Sihan Guo, Ruimin Wang, Jiewei Hu, Liping Sun, Xinru Zhao, Yufeng Zhao, Dong Han, Shuqun Hu

**Affiliations:** ^1^School of Life Sciences, Jiangsu Provincial Institute of Health Emergency, Xuzhou Medical University, Xuzhou, China; ^2^Neurobiology Institute, School of Public Health, North China University of Science and Technology, Tangshan, China

**Keywords:** cognitive function, NLRP3 inflammasome, photobiomodulation, neurogenesis, global cerebral ischemia

## Abstract

Our recent study revealed that photobiomodulation (PBM) inhibits delayed neuronal death by preserving mitochondrial dynamics and function following global cerebral ischemia (GCI). In the current study, we clarified whether PBM exerts effective roles in endogenous neurogenesis and long-lasting neurological recovery after GCI. Adult male rats were treated with 808 nm PBM at 20 mW/cm^2^ irradiance for 2 min on cerebral cortex surface (irradiance ∼7.0 mW/cm^2^, fluence ∼0.8 J/cm^2^ on the hippocampus) beginning 3 days after GCI for five consecutive days. Cognitive function was evaluated using the Morris water maze. Neural stem cell (NSC) proliferation, immature neurons, and mature neurons were examined using bromodeoxyuridine (BrdU)-, doublecortin (DCX)-, and NeuN-staining, respectively. Protein expression, such as NLRP3, cleaved IL1β, GFAP, and Iba1 was detected using immunofluorescence staining, and ultrastructure of astrocyte and microglia was observed by transmission electron microscopy. The results revealed that PBM exerted a markedly neuroprotective role and improved spatial learning and memory ability at 58 days of ischemia/reperfusion (I/R) but not at 7 days of reperfusion. Mechanistic studies revealed that PBM suppressed reactive astrocytes and maintained astrocyte regeneration at 7 days of reperfusion, as well as elevated neurogenesis at 58 days of reperfusion, as evidenced by a significant decrease in the fluorescence intensity of GFAP (astrocyte marker) but unchanged the number of BrdU-GFAP colabeled cells at the early timepoint, and a robust elevation in the number of DCX-NeuN colabeled cells at the later timepoint in the PBM-treated group compared to the GCI group. Notably, PBM treatment protected the ultrastructure of astrocyte and microglia cells at 58 days but not 7 days of reperfusion in the hippocampal CA1 region. Furthermore, PBM treatment significantly attenuated the GCI-induced immunofluorescence intensity of NLRP3 (an inflammasome component), cleaved IL1β (reflecting inflammasome activation) and Iba1, as well as the colocalization of NLRP3/GFAP or cleaved IL-1β/GFAP, especially in animals subjected to I/R at 58 days. Taken together, PBM treatment performed postischemia exerted a long-lasting protective effect on astrocytes and promoted endogenous neurogenesis in the hippocampal CA1 region, which might contribute to neurological recovery after GCI.

## Introduction

Pyramidal neuronal damage and subsequent cognitive defects are the major causes of disability in cardiac arrest patients (Moulaert et al., 2009). Global cerebral ischemia (GCI) induced by four-vessel occlusion perfectly mimics cardiac arrest, resulting in hippocampal CA1 neuronal death and delayed neurological deficits (Pulsinelli et al., 1982). Therefore, GCI animal models in rats or mice are widely used to determine molecular mechanisms and explore effective strategies. Unfortunately, except for therapeutic hypothermia, there are not yet effective therapies for preserving cognitive function after GCI. A recent meta-analysis failed to find a strong benefit of therapeutic hypothermia on survival or neurological outcome (Harukuni and Bhardwaj, 2006; Nielsen et al., 2011; Lindsay et al., 2018). Therefore, new therapeutic strategies for protecting the brain and promoting neurological recovery after cardiac arrest are urgently needed.

To our knowledge, brain injuries due to brain trauma, ischemic stroke, and transient GCI can induce prodeath signaling pathways due to oxidative stress, Ca^2+^ overload, excitotoxicity, and neuroinflammation, leading to delayed neuronal death and neurological dysfunction. Meanwhile, these brain injuries also induce inherent prosurvival reactions for self-defense and self-repair. For example, brain injury increases NSC/neural progenitor cell (NPC) proliferation followed by production of new neurons in an attempt to replenish lost or damaged cells (Marques et al., 2019). Using a mouse model of nest-green fluorescent protein reporter, a previous study revealed that endogenous NSC proliferation was increased by 120% 2 days after a traumatic brain injury (Hong et al., 2016). In an ischemic stroke model, endogenous NSC proliferation in the ischemic cortex peaks on day 2 and returns to basal levels within 6 days after stroke (Luo et al., 2009). Furthermore, in the subventricular zone (SVZ), a well-known NSC niche, the number of bromodeoxyuridine (BrdU)-labeled cells is robustly increased as early as 2 days, sustained through 4 days, and then returning to basal levels (Luo et al., 2009). A succession of studies by Tonchev’s group demonstrated that global cerebral ischemia in monkeys increases progenitor proliferation in the SVZ, subgranular zone (SGZ) of the dentate gyrus (DG), and even in the temporal neocortex at early postischemic time points during the first 15 days after ischemia (Tonchev et al., 2003a,b). Notably, newborn neurons are observed after ischemia in the hippocampal CA1 sector, the region most sensitive to ischemia (Rietze et al., 2000; Nakatomi et al., 2002), which is closely related to cognitive function. Unfortunately, several studies have revealed that almost all newborn hippocampal neurons die within days or weeks after induction of these processes (Tonchev and Yamashima, 2006; Jablonska and Lukomska, 2011; McAllister et al., 2020). Additionally, there is increasing evidence that chronic neurodegenerative diseases, such as Alzheimer’s disease (AD) and Parkinson’s disease (PD), cause significant decreases in inherent NSC proliferation and neurogenesis (Radad et al., 2017; Babcock et al., 2021). Therefore, targeting the survival of endogenous NSCs may extend the treatment window and have long-lasting beneficial effects for restoring neurobiological outcomes after brain injuries. Indeed, a recent review listed many endogenous neurogenesis-enhancing drugs, such as the P53 inhibitor pifithrin-α (PFT-α), trophic factors, and dietary supplementation, that can increase endogenous NSC proliferation, attenuate secondary cell death, and improve neurobiological functions after ischemic stroke and trauma brain injury (TBI) (Wu et al., 2017).

Recently, photobiomodulation (PBM), initially referred to as “low-level laser/light therapy (LLLT), has been attracting increasing interest in central nervous system disorders. Emerging evidence from our group and others demonstrated that PBM is promising for its many benefits, such as antioxidative, antiapoptotic, anti-inflammatory, and proneurogenic effects, mediating blood flow and the intracellular calcium response to brain injuries (Tucker et al., 2018; Wang et al., 2019; Bathini et al., 2020; Ravera et al., 2021). More importantly, PBM can activate signaling pathways and transcription factors that cause changes in protein expression, exerting long-lasting beneficial effects (Ando et al., 2011; de Freitas and Hamblin, 2016). In an ischemic stroke model, PBM is a promising treatment for inhibiting inflammasome activity (Lee et al., 2017), attenuating levels of the proinflammatory factors IL-1β and TNFα, and exerting long-lasting neuroprotective roles (Vogel et al., 2021). In traumatic brain injury animal model, transcranial low-level laser therapy not only enhances learning, memory, and neuroprogenitor cells in DG and SVZ regions of mice, but also increases brain derived neurotrophic factor (BDNF) and synaptogenesis (Xuan et al., 2014, 2015). Overall, regardless of whether the brain suffers from acute damage (stroke, traumatic brain injury, and global cerebral ischemia) or degenerative disease (dementia, Alzheimer’s disease, and Parkinson’s disease), all of these seemingly diverse conditions can be beneficially affected by applying low-level light intervention with the advantages of greater safety, lower cost, and better suitability for home use. Due to the growing interest in PBM use in the brain, we herein established a global cerebral ischemia model in adult male rats and, for the first time, investigated the roles and mechanisms of PBM treatment performed postischemia on NSC proliferation, neurogenesis and functional recovery.

## Materials and Methods

### Antibodies and Animals

The following primary antibodies were used, including NeuN (Millipore Biotechnology, MAB377, RRID: AB_2298772), BrdU (GeneTex, GTX28039, RRID: AB_385364), DCX (SCBT, sc271390, RRID: AB_10610966), GFAP (Abcam, ab53554, RRID: AB_880202), GFAP (SCBT, sc-33673, RRID: AB_627673), NLRP3 (Abcam, ab4207, RRID: AB_955792), cleaved IL1β (CST, #83186, RRID: AB_2800010), and Iba1 (Abcam, ab5076, RRID: AB_2224402).

All procedures performed in this study were approved by the Institutional Animal Care and Xu Zhou Medical University (Assurance No. 201505w001) and were conducted in accordance with the guidelines of the National Natural Science Foundation of China for animal research. Adult male Sprague-Dawley rats (3–4 months old) were housed in a temperature-controlled (22–24°C) room with freely available water and food, and ZT (zeitgeber time) was set on a 14/10 h cycle (ZT0-8:00, ZT12-22:00). The animal models including GCI model and PBM-treatment were performed in the morning in the animal model preparation room. Behavioral tests were carried out between 6:00–9:00 pm in the soundproof room of neurobehavioral laboratory. Experimental animals were sacrificed and all tissue samples were obtained in the morning.

### GCI and PBM Treatment

All surgeries were performed under isoflurane anesthesia (2–4%), and a dose of sustained release buprenorphine (Bup-SR) (0.3–1.2 mg/kg) was subcutaneously administered every 48–72 h to minimize postoperative pain. Rats were randomly assigned to the following groups: sham operation (Sham), ischemia reperfusion (I/R), photobiomodulation (PBM) treatment (I/R + PBM), and Sham with PBM treatment (Sham + PBM). The I/R group was further divided into I/R 3, 7, 28, and 58 days groups. Animals that exhibited no behavioral defects were used in this study. In order to reduce bias in the study, a double-blind procedure was carried out in which the experiments were performed by blinding investigators and statistical analysis was blindly worked out by the authors.

The global cerebral ischemia (GCI) animal model was induced by four-vessel occlusion as described in our previous study (Bai et al., 2020). In brief, rats were fixed on the stereotaxic instrument, and vertebral arteries were permanently occluded by electrocautery. Then, the bilateral common carotid arteries (CAAs) were exposed, and the incision was sutured. One day later, the bilateral common carotid arteries of the rats were re-exposed and clipped with an aneurysm for 12 min. Rats with an absence of the correctional reflex within 30 s that were unresponsive to light, resulting in dilated pupils during ischemia, were identified as successfully modeled. Rectal temperature was maintained at approximately 36.5–37.5°C during the procedure using an incubator. For sham-operated animals, all rats were treated exactly as for ischemic animals except that the CCA was not clamped. A total of 95 rats were utilized in this study. From all 95 animals, 8 rats died in ischemia reperfusion, and 11 rats were eliminated from further experiment due to not meeting the established criteria for evidence of successful GCI. The animal numbers for each experimental paradigm were indicated in the figure legends.

For PBM treatment, a diode laser (808 nm, model 808M100, Dragon Lasers) was used in the study, performed as our previously described (Wang et al., 2019). Briefly, laser penetration through tissues was measured with isolated rat samples of skin, skull, and brain tissues (cortex and hippocampus). A sample holder was made from black Plexiglas^R^ with an 8 mm hole in its center. To evaluate the influence of the power in the penetration of light through different tissue samples, the laser source was located 15 cm away from the sample and two photodetectors let the simultaneous measurement of light power transmitted through the different tissues we placed on the holder, and a portion of the incident light. We measured the power without any sample between the light source and the detector (laser in mW/cm^2^ across of air), and then detected the percentage of transmitted light when samples were placed on the system (laser in mW/cm^2^ across of tissue). Based on the preliminary experiment of light penetration on cerebral tissues, we selected the laser power output at the tip of the fiberoptic probe (in contact with shaved skin) delivered a power density of 20 mW/cm^2^ to the cerebral cortical surface. The laser irradiation was delivered for 2 min at this power density (20 mW/cm^2^) so that the fluence at the cortical surface was 2.4 J/cm^2^ and at the hippocampus was ∼0.8 J/cm^2^. The dose of laser power (fluence) was calculated by total irradiated time (second) × power output (mW/cm^2^)/1,000 and expressed as J/cm^2^. A laser power meter (#FM33-056, Coherent Inc., United States) was applied to monitor the power output density.

Followed by slightly anesthetization using isoflurane, the rats were fixed in transparent bags, the laser beam transcranially focused on ∼8 mm diameter spot covering the shaved scalp (centered at 3 mm posterior to the eye and 2 mm anterior to the ear). The PBM treatment was begun at 3 days of reperfusion after GCI for 2 min, once daily for 5 consecutive days. Sham-operated controls underwent the same procedure as the laser-treated group but did not receive actual laser treatment.

### 5-Bromo-2′-Deoxyuridine (BrdU) Injection

BrdU (CAS# 19-160, Sigma-Aldrich) was diluted in 0.1 M PBS to make a sterile solution of 10 mg/mL. The rats received intraperitoneally BrdU injections (50 mg/kg body weight) twice daily over 7 consecutive days, starting reperfusion 1 day after GCI, and were, respectively, killed at I/R 7 days (2 h after the last BrdU injection), and I/R 58 days after GCI.

### Morris Water Maze (MWM) Test

The Morris water maze test was performed on days 7–9, and 56–58 as previously described (Wang et al., 2019). In brief, a circular pool filled with water (1.2 m in diameter, 35 cm in height) containing a platform concealed below the surface (2.0 cm) was used. The pool was equally divided into four quadrants. During the adaptive training procedure, rats were randomly placed in one of the quadrants facing the wall of the pool and allowed to swim for a maximum time of 90 s until they discovered the fixed platform. If the rat was unable to find the platform within 90 s, it was gently guided to the platform and allowed to rest on the platform for 20 s. The test was repeated four times a day starting from different quadrants with a 2 min intertrial interval for 3 consecutive days. Six hours after the final experiment, the hidden platform was removed, and rats were placed in the pool in the same quadrant and allowed to explore for 60 s. The time spent in the quadrant that previously contained the platform was measured to evaluate the level of spatial reference memory for the given task. All behavioral tracks from the trials were recorded and analyzed using video tracking software, and the resultant data were statistically analyzed as described below.

### Immunofluorescence Staining and Confocal Microscopy

Immunofluorescence (IF) staining was performed as previously described (Bai et al., 2020). In brief, cardiac perfusion was performed in rats using 0.9% saline solution. The brains were rapidly isolated on an ice plate and subsequently fixed in 4% paraformaldehyde overnight at 4°C. The brain tissues were completely dehydrated with 30% sucrose, and make in continuous coronal frozen sections (25 μm) in the coronal plane of the dorsal hippocampus level at 2.5–4.5 mm posterior from bregma (∼100 sections per brain). To prevent double counting of cells, every fifth section was collected and used for staining. After washing in 0.1 M phosphate-buffered saline (PBS) for 30 min, sections were permeabilized with 0.4% Triton X-100-PBS for 2 h and incubated with 10% normal donkey serum for 1 h at room temperature before being incubated overnight at 4°C with primary antibodies. The following primary antibodies were used in the current study: NeuN (1:300), NLRP3 (1:200), cleaved IL-1β (1:500), and Iba1 (1:1,000). For double IF staining of BrdU with GFAP, a set of sections containing every sixth slice were denatured with 1 N HCl in a 37°C water bath for 20 min. Sections were then washed with 0.1% Triton X-100-PBS three times and blocked in 10% normal donkey serum at room temperature for 1 h. Sections were then incubated with anti-BrdU antibody (1:500) and GFAP (1:1000) overnight at 4°C. After washing, the sections were incubated with Highly Cross-Adsorbed Alexa Fluro IgG second antibodies (Thermo Fisher Scientific) for 1 h at room temperature. In detail, donkey anti-mouse 488 nm (A-21202, RRID: AB_141607, Excitation 488 nm and Emission 525 nm) was for NeuN or BrdU staining; donkey anti-goat 488 nm (A32814, RRID: AB_2762838) was for Iba1 staining; donkey anti-mouse 488 nm (A-21202, RRID: AB_141607) and donkey anti-goat 594 nm (A-11058, RRID: AB_2534105, Excitation 566 nm and Emission 610 nm) were for BrdU and GFAP double staining; donkey anti-goat 488 nm (A32814, RRID: AB_2762838) and donkey anti-mouse 594 nm (A-21203, RRID: AB_141633) were for DCX and NeuN double staining; donkey anti-mouse 488 nm (A-21202, RRID: AB_141607) and donkey anti-goat 594 nm (A-11058, RRID: AB_2534105) were for GFAP and NLRP3 double staining; donkey anti-rabbit 488 nm (A-21206, RRID: AB_2535792) and donkey anti-goat 594 nm (A-11058, RRID: AB_2534105) were for Cleaved-IL1β and GFAP double staining. Following three final washes for 10 min each, sections were mounted and cover slipped in Vectashield mounting medium with DAPI (H-1200; Vector Laboratories, Inc., CA, United States). All confocal images were captured on an FV1000 confocal laser microscope (Olympus) and digital imaging software (FV10-ASW 1.5 Viewer). For quantitative analysis, the number of surviving neurons per 250 μm length of medial CA1 pyramidal cell layer was counted bilaterally in five representative sections per animal. Furthermore, the fluorescence intensity of the targeting protein was normalized as the percent change compared to the control group as indicated in the figures. If necessary, colocalization was analyzed using Fiji software (version 1.52q).

### Ultrastructural Morphology of Astrocytes and Microglia Cells the Hippocampal CA1 Region by Transmission Electron Microscopy (TEM)

According to a previous description (Zhou et al., 2011), the hippocampal CA1 region was cut into small tissue blocks (1 mm^3^) and fixed in 2.5% glutaraldehyde and 2.0% paraformaldehyde for 4 h in 0.1 mol/l phosphate buffer at 4°C. After fixation with 2% osmium tetroxide for 30 min, tissues were dehydrated in a series of graded ethanol solutions. Subsequently, ethanol was substituted with propylene oxide, and tissues were then embedded in Epon 812. Ultrathin sections (70 nm) were mounted on 200-mesh copper grids that were counterstained with uranyl acetate (30 min) followed by lead citrate for 10 min. Finally, the copper grids were washed with PBS and distilled water, and the ultrastructure was observed by transmission electron microscopy (H Hitachi-7650).

### Statistical Analysis

Data were analyzed by one- or two-way ANOVA followed by the Student–Newman–Keuls program using SigmaStat 3.5 software and are presented as the mean ± SEM. Differences were considered significant at ^∗^, ^∗∗^, and ^∗∗∗^, which denote *P* < 0.05, *P* < 0.01, and *P* < 0.001, respectively. The statistical figures were generated using GraphPad Prism 8 software.

## Results

### PBM Treatments Preserves CA1 Neurons After GCI

**FIGURE 1:**
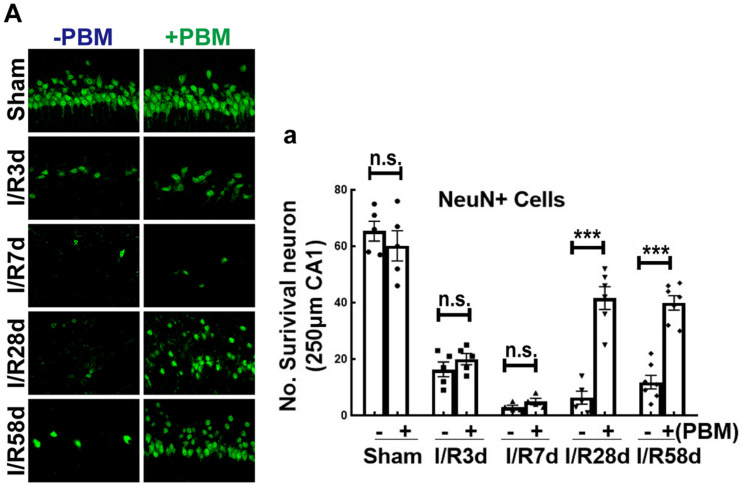
The effect of photobiomodulation (PBM) treatment on neuronal survival in the hippocampal CA1 region following global cerebral ischemia (GCI). **(A)** Representative images of NeuN staining (green, reflecting surviving neurons) in the indicated groups and quantification of NeuN+ cells **(A-a)** in the 250 μm hippocampal CA1 region, showing the beneficial effects of PBM on the 28th and 58th days but not on the 3rd or 7th day after GCI (*n* = 5–7 in each group). ***P < 0.001 between the two groups. n.s., no significant difference. Scale bar: 50 μm; magnification: 40×.

Transient global cerebral ischemia in gerbil for 15 min causes significant neuronal loss 2 days after reperfusion in the hippocampal CA1 region (Lee et al., 2019). On the other hand, cerebral ischemia can also activate endogenous programs of NSC proliferation, such as gliosis and neurogenesis, beginning 4 days after reperfusion in the hippocampal CA1 region, while newborn cells become gradually injured following reperfusion after GCI (Wang et al., 2005; Tonchev and Yamashima, 2006). To determine whether PBM treatment protects newborn cells and favors recovery of cognitive outcome after GCI, we performed PBM treatment beginning 3 days after reperfusion for 5 consecutive days and evaluated neuronal survival in the hippocampal CA1 region following the time course after GCI (I/R 3, 7, 28, and 58 days). Immunofluorescence staining for NeuN was used to assess neuronal survival. Quantification of the results is provided in Figure 1A,a. As expected, the number of surviving neurons (NeuN-positive cells, green) was significantly decreased at all four timepoints (I/R 3, 7, 28, and 58 days) and reached a trough I/R 7 days after GCI compared to sham group animals. Intriguingly, at later timepoints (I/R 28 and 58 days), PBM-treated animals exhibited a greater number of NeuN+ cells than early timepoints (I/R 3 and 7 days), and PBM significantly increased NeuN+ cells compared to I/R animals at the same timepoints. These results indicate that PBM treatment for 5 consecutive days postischemia exerted long-lasting, effective protection against ischemic insult, likely involving attenuation of cell loss in the hippocampal CA1 region.

### PBM Treatment Only Improves Cognitive Function at Later Timepoints After GCI

**FIGURE 2:**
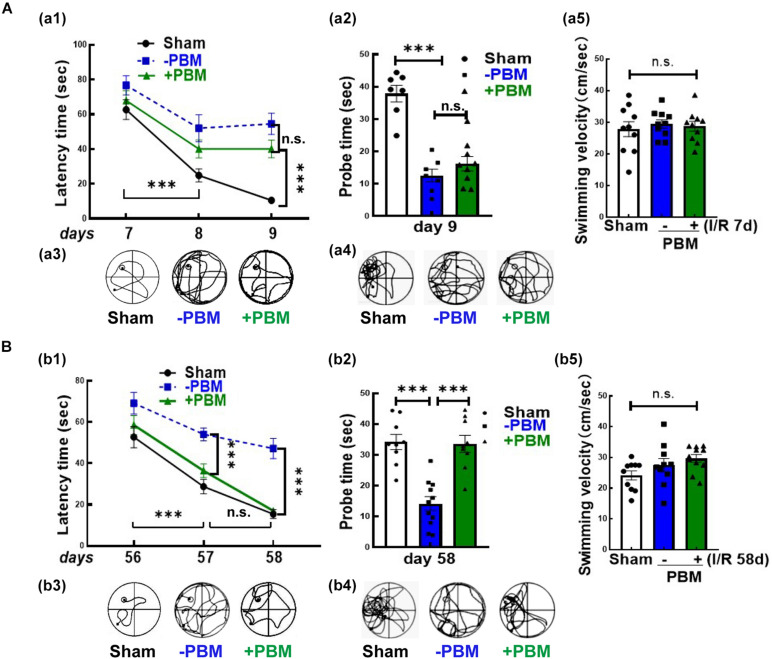
Photobiomodulation treatment only improves functional outcome at the later time point (56–58 days) but not at the early time point (7–9 days) after GCI. Spatial learning/memory test in the Morris water maze 7–9 days **(A)** and 56–58 days **(B)** after GCI. **(a1,b1)** Latency trial and **(a2,b2)** probe trial; swimming paths of the rats from latency trials and probe trials on day 9 **(a3,a4)** and day 58 **(b3,b4)**; swimming velocity of probe trials on day 9 **(a5)** and day 58 **(b5)** after GCI (*n* = 10–11 in each group). ^∗∗∗^*P* < 0.001 between the two groups. n.s., no significant difference. GCI, global cerebral ischemia; PBM, photobiomodulation.

We next examined whether PBM treatment preserved hippocampal-dependent working memory using the Morris water maze 7–9 and 56–58 days after GCI reperfusion. Figures 2A,B show the results of the latency trial (reflecting learning ability) and probe trial (reflecting memory ability) at early and later timepoints of reperfusion, respectively. We found that all non-ischemic animals spent less time (latency time) finding the target platform and increased the time (probe time) spent exploring the quadrant where the platform had been (Figures 2Aa1,a2,Bb1,b2). PBM treatment only improved cognitive function at the later timepoint but not at the early timepoint, as evidenced by PBM-treated animals exhibiting a significantly decreased latency time and significantly enhanced probe time at the later timepoint but no significant change at the early timepoint (Figures 2Aa1,a2,Bb1,b2). Representative tracings indicating sample swim paths of the rats from the latency trial and probe trial are shown in Figures 2Aa3,a4,Bb3,b4. The escape velocity of all animals showed no significant changes both in latency and probe trails (Figures 2Aa5,Bb5). Taken together, our findings suggest that short-term PBM treatment postischemia may provide a long-lasting beneficial effect for cognitive recovery after GCI, but mechanistic studies need to be performed.

### PBM Treatment Elevates Endogenous NSC Proliferation Following GCI

**FIGURE 3:**
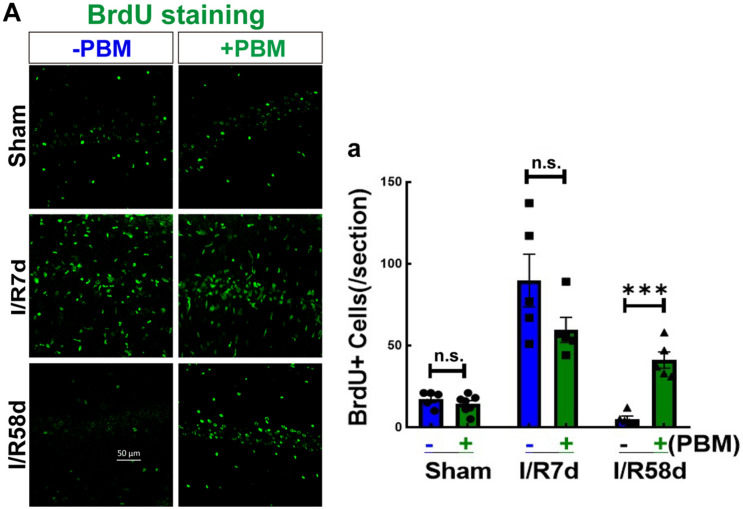
Photobiomodulation elevates NSC proliferation at I/R 58 days reperfusion after GCI in the hippocampal CA1 region. Hippocampal coronal sections from sham and I/R 7 days and I/R 58 days rats treated with or without PBM were used to detect NSC proliferation (BrdU+). **(A)** Representative images of IF staining for BrdU (green) in the hippocampal CA1 regions and **(a)** quantification of BrdU+ cells in the sections. n.s., no significant difference (*n* = 5–7 in each group). ^∗∗∗^*P* < 0.001 between the two groups. Scale bar: 50 μm; magnification: 40×.

It is well known that neurogenesis can replenish lost or damaged cells (Marques et al., 2019), which contributes to neurological repair after brain injury (Parpura et al., 2012). However, it is unknown whether PBM treatment improves neurogenesis following GCI. The traditional view is that neurogenesis occurs in two discrete regions, the subventricular zone (SVZ) of the olfactory bulb and the subgranular zone (SGZ) of the hippocampal dentate gyrus (DG). Herein, we examined the effects of PBM on the NSC proliferation program in the hippocampal CA1 region, which is the region most vulnerable to GCI. We first assessed the effect of PBM treatment on NSC proliferation following GCI by BrdU staining. As shown in Figure 3A,a, compared to the sham group, the number of BrdU+ cells (green, reflecting NSC proliferation) was dramatically enhanced at I/R 7 days and then markedly decreased over time (I/R 58 days) after GCI. PBM treatment did not significantly affect the number of BrdU+ cells in the hippocampal CA1 region of I/R 7 days or non-ischemic animals. Notably, PBM treatment markedly enhanced BrdU+ cells in the hippocampal CA1 region 58 days after reperfusion compared to those in the I/R 58 days group. These results indicate that PBM treatment may prevent cell loss by enhancing NSC proliferation at later time points after GCI in the hippocampal CA1 region.

### PBM Promotes Neurogenesis at the Later Timepoint After GCI Reperfusion

**FIGURE 4:**
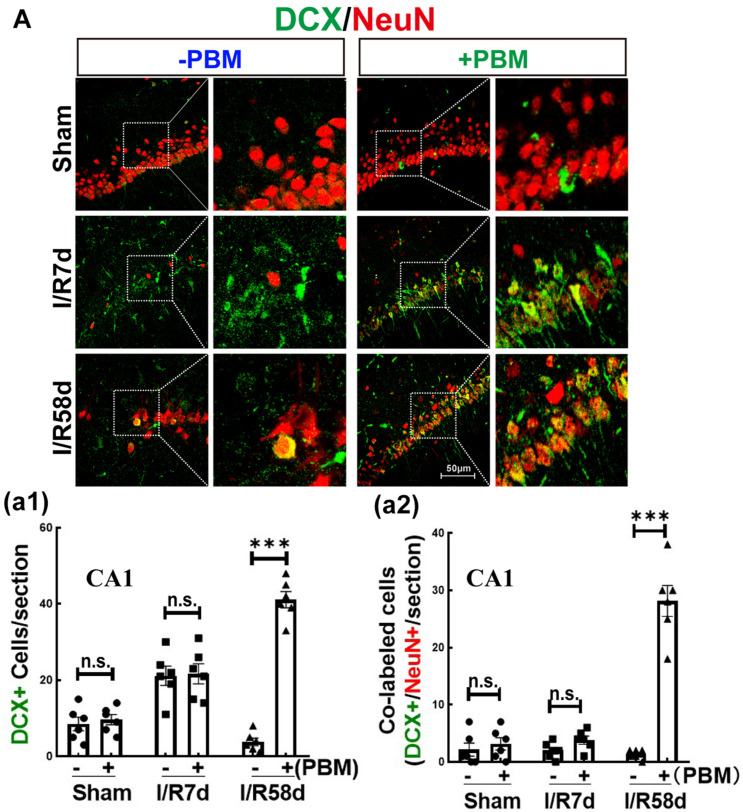
Photobiomodulation promotes proliferating NSCs toward neurons 58 days after GCI reperfusion in the hippocampal CA1 region. Hippocampal coronal sections from sham, I/R 7 days and I/R 58 days rats treated with or without PBM were used to detect neurogenesis by IF staining. DCX is a marker of newborn immature migrating neurons, and NeuN is a marker for surviving neurons. Colabeled cells reflect proliferating neurons. **(A)** Representative images of double IF staining for DCX (green) and NeuN (red) in the hippocampal CA1 region. Quantification of DCX+ cells **(a1)** and DCX+/NeuN+ colabeled cells (yellow) **(a2)** in the hippocampal CA1 region (*n* = 5–7 in each group). ns, no significant difference. ^∗∗∗^*P* < 0.001 between the two groups. Scale bar: 50 μm; magnification: 40×.

Neural stem cells possess multipotential to differentiate into both neuronal and glial subtypes, such as astrocytes, microglia and oligodendrocytes (Rolando et al., 2016). To further assess NSC fate induced by GCI with or without PBM, we next performed double IF staining for NeuN (reflecting mature neurons) and DCX (reflecting newborn/immature neurons). Figure 4A-a1 shows that there was no significant change in the number of DCX+ cells (green) in the CA1 region in the sham or I/R 7 days groups with or without PBM treatment. Compared to sham controls, DCX+ cells significantly increased at I/R 7 days and decreased at I/R 58 days after reperfusion, but PBM treatment robustly enhanced DCX+ cells in the hippocampal CA1 region compared to I/R 58 days without PBM treatment. Only I/R 58 days PBM-treated animals displayed significantly enhanced numbers of DCX-NeuN colabeled cells, and there were no significant changes in the other groups (Figure 4A-a2). These results indicate that PBM treatment drives endogenous NSCs toward neuronal lineages in the hippocampal CA1 region only at later time points after GCI. Based on these results, we conclude that neurogenesis in the hippocampal CA1 region may contribute to neurological functional recovery caused by PBM treatment at I/R 58 days after GCI, while further studies will be necessary to elucidate its mechanism.

### PBM Suppresses Extensive Activation of Astrocytes in the Hippocampal CA1 Region Following GCI

**FIGURE 5:**
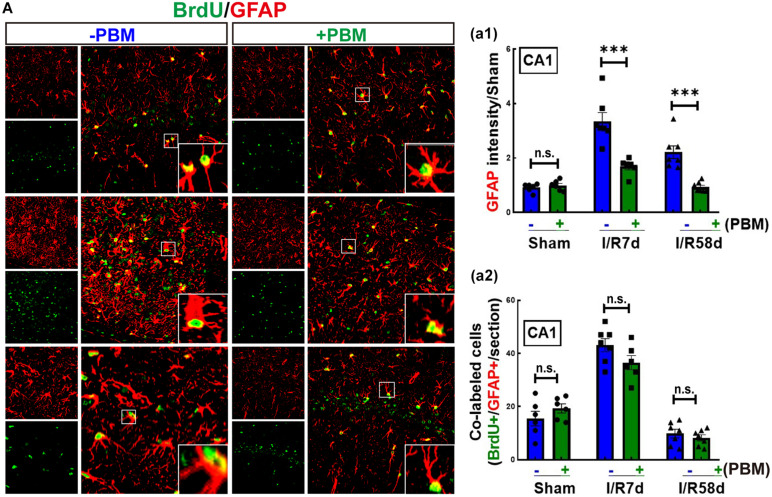
Global cerebral ischemia enhances astrogliosis and reactive astrocytes 7 days after GCI reperfusion, and PBM suppresses astrocyte activation in both I/R 7 days and I/R 58 days animals. **(A)** Representative images of double IF staining for BrdU (green) and GFAP (red, an astrocyte marker). **(a1)** Quantification of GFAP fluorescence intensity (reflecting astrocyte activation), showing that PBM treatments attenuated reactive astrocytes compared to the I/R groups at the same timepoint in the hippocampal CA1 region. **(a2)** Quantification of the number of BrdU/GFAP colabeled cells (reflecting astrocytic regeneration/astrogliosis), showing that PBM had no significant effect on any of the indicated groups (*n* = 6–7). ^∗∗∗^*P* < 0.001. n.s., no significant difference. scale bar 50 μm; magnification 40×.

Neural stem cells can also differentiate into glial cells, which mediate the microenvironment to determine the destiny of newborn neurons (Koehl, 2015). We therefore performed double IF staining for BrdU and GFAP (astrocyte marker) to examine astrocytic glial regeneration and activation. As shown in Figure 5A-a1, GCI significantly enhanced the fluorescence intensity of GFAP (reflecting astrocyte activation) in the hippocampal CA1 region compared to that of the sham group, and PBM treatment markedly suppressed this enhancement at both time points. The morphology of astrocytes in the GCI groups presented far fewer and shorter branches with hypertrophic bodies than that of PBM-treated animals. Furthermore, BrdU+/GFAP+ colabeled cells were quantified in the hippocampal CA1 region, which reflects NSC differentiation toward astrocytes (astrogliosis). PBM treatment did not exhibit a significant effect on the number of BrdU+/GFAP+ colabeled cells; however, I/R 7 days either with or without PBM treatment dramatically elevated the number of BrdU+/GFAP+ colabeled cells compared to the sham group, and I/R 58 days led to a significant reduction in colabeled BrdU+/GFAP+ cells compared to the I/R 7 days group (Figure 5A-a2). Taken together, our findings demonstrate that PBM treatment suppresses extensive activation of astrocytes, which is likely a major cause of locally enhanced neurogenesis in the hippocampal CA1 region at the later time point after GCI.

### PBM Treatment Attenuates Astrocyte NLRP3 Inflammasome and Inflammatory Impairment in the Hippocampal CA1 Region After GCI

**FIGURE 6:**
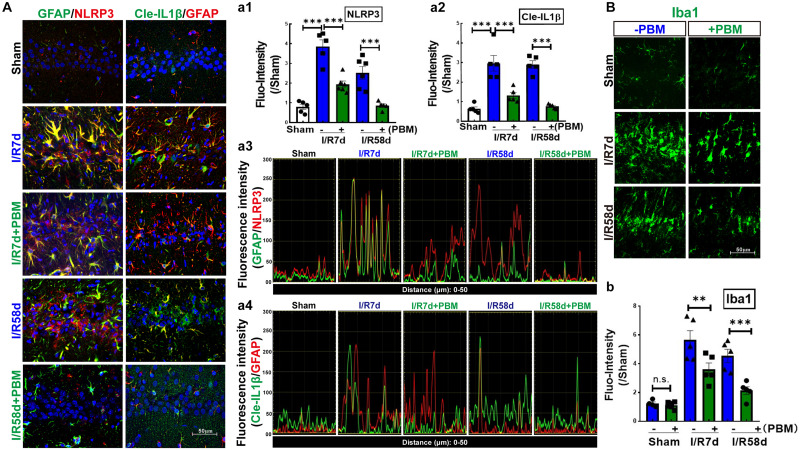
Photobiomodulation treatment inhibits GCI-induced expression of NLRP3 and activates IL-1β in the hippocampal CA1 region following GCI. **(A)** Representative images of double IF staining of GFAP/NLRP3 and Cle-IL-1β/GFAP in the indicated groups. Fluorescence intensity quantification of NLRP3 **(a1)** or Cle-IL-1β **(a2)**. Data are expressed by the ratio of fluorescence intensity of the section compared to sham. Colocalization between the two stained proteins for GFAP/NLRP3 **(a3)** and Cle-IL-1β/GFAP **(a4)**. **(B)** Representative images of IF staining for Iba1 (a microglial marker) and fluorescence intensity quantification **(b)** (*n* = 5–6 in each group). ^∗∗^*P* < 0.01; ^∗∗∗^*P* < 0.001; scale bar 50 μm; magnification 40×.

Upon inflammasome activation, NLRP3 assembles its adaptor ASC and produces a multiprotein complex with pro-caspase-1, which leads to caspase-1 activation and IL-1β maturation (cleaved IL-1β). Thus, we hypothesized that NLRP3 inflammasome activation, particularly in damaged astrocytes, may represent an important pathway in early-stage impairment after GCI; therefore, inhibition of the NLRP3 pathway by PBM treatment favors neurological recovery. To confirm our hypothesis, we examined protein expression of NLPR3 and cleaved IL-1β in the hippocampal CA1 region following GCI using IF staining analysis. Representative images of double IF staining for NLRP3 (red) and GFAP (green), cleaved IL-1β (Cle- IL1β, green) and GFAP (red) are shown in Figure 6A. These results indicate that the immunofluorescence intensity of NLRP3 (Figure 6A-a1) and Cle-IL1β (Figure 6A-a2) was robustly enhanced in both the I/R 7 days and I/R 58 days groups and strongly colocalized in the I/R 7 days group compared to the sham control (Figures 6A-a3,a4). We did not observe strong colocalization of NLPR3/GFAP or Cle-IL1β/GFAP in the I/R 58 days group, although the immunofluorescence intensity of NLPR3 or Cle-IL1β was still strong in these groups. PBM treatment significantly mitigated the I/R-induced enhancement of NLRP3 or Cle-IL1β and their colocalization with GFAP (Figures 6A-a3,a4). Finally, we performed IF staining for Iba1, a microglial marker, to detect inflammatory injury in the hippocampal CA1 region following GCI. Similarly, we found that the immunofluorescence intensity of Iba1 exhibited a similar pattern, with a robust elevation in Iba1 reactivity expression in the I/R groups compared to the sham group. In I/R 58 days animals, amoeboid Iba1+ cells nearly entirely occupied the hippocampal CA1 region, showing a similar pattern to NLRP3 and Cle-IL-1β. Importantly, PBM treatment attenuated I/R-induced enhancement in Iba1 protein levels (Figure 6B-b). Overall, our findings suggest that PBM treatment suppresses NLPR3 inflammasome activation in astrocytes at the early stage of reperfusion, which prevents later-phase inflammatory impairment following GCI.

### PBM Treatment Preserves the Ultrastructure of Astrocytes and Microglia Cells in the Hippocampal CA1 Region Following GCI

**FIGURE 7:**
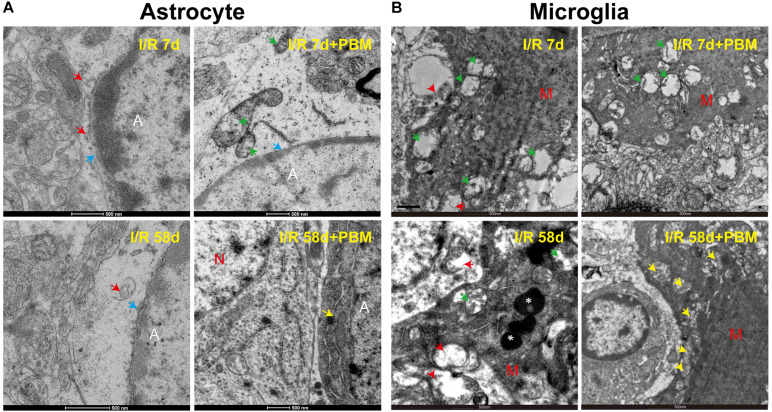
The effects of PBM on astrocyte and microglia ultrastructure in the hippocampal CA1 region of I/R 7 days and I/R 58 days animals. Representative electron microscopy images show the ultrastructure of astrocytes **(A)** and microglia cells **(B)** in the indicated groups. Yellow arrows: integrity mitochondria; red arrows: disaggregated mitochondria; green arrows: mitochondria with broken cristae; white star: lysosome; blue arrows: nuclear membrane. A, astrocyte; M, microglia; N, neuron.

Recently, astrocyte-microglia communication has attracted increased attention because it directly mediates the surrounding microenvironment to determine the fate of resident precursor cells and impacts tissue repair and recovery after brain injury (Cope and Gould, 2019; Greenhalgh et al., 2020). Given that PBM significantly affects the morphology and activation of astrocytes and microglia cells following GCI, we performed transmission electron microscopy (TEM) to observe their ultrastructural changes in the hippocampal CA1 region in response to PBM treatment. Under physiological condition, astrocytes have pale nuclei that are usually regular in shape and their cytoplasm is also pale. A characteristic of astrocytes is that they are sometimes partially surrounding synapsing axons, spines and dendrites, and their processes are extending into the surrounding neuropil. However, response to ischemic injury or neurodegenerative diseases, astrocytic ultrastructure shows unhealthy features, such as astrocytic swelling, nuclear shrinkage, chromatin condensation, organelles (mitochondria and endoplasmic reticulum) damage/loss, and translucence of matrix (gliofilament disappearance) (Kwon et al., 2009). As shown in Figure 7A, a significant amount of chromatin condensation was observed on top of the nuclear membrane, and the nuclear membrane partly disappeared in the I/R 7 days group, indicating overactivation. In the I/R 58 days group, the cytoplasm of the astrocytes appeared completely empty, with disappeared organelles and gliofilaments. PBM treatment reduced the damage to I/R 58 days animals, showing distinct normalization in the ultrastructural picture with pale nuclei and plumpy cytoplasm (intact mitochondria, yellow arrow), as well as adhering to a relatively normal neuron (N) (Figure 7A). Meanwhile, Figure 7B shows that in the GCI groups (I/R 7 days and I/R 58 days), the microglia ultrastructure exhibited severe mitochondrial impairment, characterized by numerous vacuolated mitochondria with some partially broken cristae (green arrow) and even disaggregation (red arrow). In striking contrast, we found that sections from I/R 58 days in PBM-treated animals clearly showed nearly normal ultrastructure of microglia cells, in which the majority of mitochondrial structure was intact with round and very few broken cristae (yellow arrow), while I/R 7 days PBM-treated animals did not experience a beneficial effect and displayed obvious mitochondrial damage. These results suggest that PBM protects astrocyte and microglia at only the later timepoint but not the early timepoint after GCI, which might contribute to the long-lasting protective and repair roles observed for cognitive outcomes.

## Discussion

The traditional view is that neurogenesis initiates in the SVZ and the SGZ, which are the major niches of NPCs and NSCs. Furthermore, brain injuries due to ischemic stroke and brain trauma promote NPCs and NSCs to migrate toward the lesion area and differentiate into neurons and glial cell (Marques et al., 2019). However, it has been reported that in a transient global ischemia animal model, selectively leading to hippocampal CA1 neuronal delayed death, ischemia-induced SGZ neurogenesis is not attributed to CA1 neuronal loss (Liu et al., 1998; Salazar-Colocho et al., 2008; Song et al., 2021). Notably, several studies have found that locally increased NSC proliferation and newborn neurons appear in the hippocampal CA1 region, although neurogenesis is very limited due to the extreme vulnerability of the CA1 region to ischemia insult (Schmidt and Reymann, 2002) and a very harsh environment, such as inflammation (Tobin et al., 2014), in which newly born hippocampal neurons die within days or weeks (Jablonska and Lukomska, 2011). Therefore, in the current study, we aimed to develop a potential strategy that promotes local NSC proliferation and direct differentiation toward neurons in the hippocampal CA1 region.

Photobiomodulation therapy has emerged as a potential novel non-invasive intervention for acute brain injuries caused by stroke, traumatic brain injury, and global ischemia (Wang et al., 2019; Dompe et al., 2020), as well as chronic neurodegenerative diseases, such as pain, Parkinson’s disease, and Alzheimer’s disease (Farfara et al., 2015; Berman and Nichols, 2019; Salehpour and Hamblin, 2020). The results of our current study demonstrate that (1) PBM treatment enhances endogenous NPC proliferation and local neurogenesis at I/R 58 days after GCI reperfusion in the hippocampal CA1 region; (2) PBM treatment not only attenuates the early inflammatory response but also maintains the NPC proliferation induced by GCI (I/R 7 days after GCI); (3) notably, PBM treatment improves neurological recovery only at the later time point (I/R 58 days) but not at the early time point (I/R 7 days) after GCI reperfusion.

In our current study, we used an 808 nm laser with a power dose of 20 mW/cm^2^. Supporting our study, Tedford et al. (2015) confirmed that 808 nm light can reach a depth in the brain of 40–50 mm (Hamblin, 2016) and that a laser power dose of 20 mW/cm^2^ significantly improves the neurological function of mice in a traumatic brain injury model (Oron et al., 2007; Oron et al., 2012). A novel discovery by our research group is the remarkable protection of hippocampal CA1 region neurons in male rats 1 week and 6 months after ischemia, along with prominent functional improvements in cognition induced by 808 nm 8 mW/cm^2^ PBM-treatment at the hippocampus tissue (Wang et al., 2019). Herein, to explore whether laser treatment altered local neurogenesis in the hippocampal CA1 region, we treated animals with PBM 3 days after reperfusion onset for 5 successive days. The reason we chose this time window for PBM treatment was based on the survival curves of hippocampal CA1 neurons following GCI from other studies (Lee et al., 2019) and our current studies. We found that NeuN-positive cells (reflecting surviving neurons) were sharply decreased at I/R 3 days and were hardly detectable at I/R 7 days and I/R 28 days after GCI compared to the sham group in the CA1 region. However, there was a slight increase in the number of NeuN+ neurons at I/R 58 days compared to I/R 7 days animals, but there was no significant difference compared to I/R 28 days after GCI in the CA1 region. Importantly, PBM treatment did not increase neuron survival at I/R 3 days or I/R 7 days; however, PBM significantly enhanced neuron survival at I/R 58 days, as evidenced by increased NeuN+ cells in the hippocampal CA1 region compared to I/R animals at the same time-point. These results suggest that PBM might improve neurogenesis rather than protect impaired neurons in the hippocampal CA1 region. To confirm this hypothesis, we next performed BrdU staining, a marker to examine the proliferation of NSCs *in situ* (Nemirovich-Danchenko and Khodanovich, 2019). Our results revealed that BrdU+ cells were dramatically elevated at I/R 7 days and then significantly decreased at I/R 58 days compared to the sham group in the hippocampal CA1 region. Intriguingly, NSC proliferation was retained at I/R 7 days and at I/R 58 days in the PBM-treated group because increased BrdU+ cells primarily occupied the CA1 region of the hippocampus. Furthermore, we observed substantial neurogenesis in the hippocampal CA1 region of I/R 58 days animals treated with PBM, as evidenced by a significant increase in DCX+/NeuN+ colabeled cells. Additionally, astrogliosis was significantly enhanced at the early time point (I/R 7 days) with or without PBM treatment, as evidenced by a dramatic enhancement in BrdU+/GFAP+ colabeled cells. Notably, extensive activation of astrocytes in the I/R 7 days group was suppressed by PBM treatment as shown by a marked decrease in the fluorescence intensity of GFAP in the PBM-treated animals compared to the I/R 7 days animals. The Morris water maze test is used to determine the function of hippocampal-dependent learning and memory (Nakazawa, 2006). Further functional studies revealed that PBM treatment improved cognitive deficits induced by GCI at the later time point but not the earlier time point. A few studies have shown that in global cerebral ischemia animal models, increased NSC proliferation starts at 3–4 days, peaks at 7–10 days and then dramatically decreases in the hippocampal DG region (Liu et al., 1998; Song et al., 2021). In contrast to these reports, our current study, for the first time, elucidates that PBM preserves endogenous proliferative NSCs induced by GCI and promotes their differentiation toward neurons in the hippocampal CA1 region. Supporting our findings, Oron et al. (2006) reveal that low level PBM performed at 24 h post-stroke exerts a significant functional benefit with an underlying mechanism possibly being induction of neurogenesis. More importantly, an excessive number of laser-treatments can temporarily delay the process of brain repair stimulated by PBM by causing temporary induction of reactive gliosis (Xuan et al., 2016).

Overactivation of astrocytes leads to the accumulation of damaged ROS-generating mitochondria, and this triggers Nod-like receptor protein 3 (NLRP3) inflammasome activity, which in turn leads to a neuroinflammatory response and neuronal impairment (Schultz et al., 2004; Jones et al., 2018). The active NLRP3 inflammasome cleaves proinflammatory IL-1β to its active form, which is a key determinant of outcome after brain injuries (Mezzasoma et al., 2016; Bai et al., 2020). Several studies have revealed that astrocytes are the predominant source of IL-1β in traumatic brain and ischemic disorders (Jones et al., 2018). Consistent with these reports, our current study revealed that GCI triggers increased NLRP3 expression at both early and later time points, and cleaved IL1β was correlated with significant enhancement in overactivated astrocytes of the hippocampal CA1 region. Furthermore, protein expression of Iba1, a marker of inflammatory injury, was persistently elevated, which perfectly mirrored changes in NLRP3 activity. Importantly, the increased NLRP3 activity and inflammation caused by GCI were suppressed by PBM treatment. PBM exerts anti-inflammatory effects in a wide range of animal models, such as acute traumatic brain injury, experimental autoimmune encephalomyelitis, spinal cord injury and wound healing (Hamblin, 2017; Xu et al., 2017). *In vitro* studies also confirmed that PBM treatment has the ability to change the phenotype of activated microglia (Fernandes et al., 2015; Hwang et al., 2015; Lim et al., 2015; Saha et al., 2016), especially at lower power doses (von Leden et al., 2013). Recent work from Zhang’s Lab. demonstrates that PBM-treatment from 2 to 8 post-stroke in rats can effectively switch an M1 microglial phenotype to an anti-inflammatory M2 phenotype, and promotes neurogenesis (Arsac and Frileux, 1988). In addition, treatment with PBM has been shown to enhance cognitive capability in normal people (Barrett and Gonzalez-Lima, 2013) and healthy animals (Gonzalez-Lima and Barrett, 2014). In acute brain injuries in humans, such as ischemic stroke and traumatic brain injury (Lampl et al., 2007; Figueiro Longo et al., 2020), as well as chronic neurodegenerative diseases, such as AD and PD (Liu et al., 2020; Hong et al., 2021), PBM intervention has likewise been shown to elevate functional cognitive recovery in both humans and animals. Based on these findings, we propose that inhibition of NLRP3 inflammasome activation and IL-1β in astrocytes of the hippocampal CA1 region could contribute, in part, to pro-neurogenesis and cognitive recovery in response to PBM treatment after GCI.

Additionally, we demonstrated that although PBM treatment attenuated the early extensive inflammatory response at I/R 7 days after GCI in the hippocampal CA1 region, the number of surviving neurons was not significantly increased, and working memory was not improved at this time point. This may be due to the unhealthy ultrastructure of glial cells, which may be caused by other damage, such as oxidative stress and endoplasmic reticulum stress. Indeed, our EM data corroborate the hypothesis that mitochondria were damaged in microglia cells and degradative subcellular organelles in astrocytes at I/R 7 days with or without PBM treatment after GCI in the hippocampal CA1 region. On the other hand, in line with our findings, increasing evidence has demonstrates that glial cells such as astrocyte, microglia and oligodendrocyte cells, act directly on neurons to protect against ischemic insults (Carmen et al., 2007; Nutma et al., 2020). However, further studies will be necessary to elucidate the precise mechanism underlying the effects induced by PBM treatment.

## Conclusion

The current study is the first to demonstrate that PBM treatment protects endogenous NSCs and promotes local neurogenesis in the hippocampal CA1 region, which is partly due to mitigating the inflammatory impairment induced by NLPR3 inflammasome activation. The current study provides a potential strategy for repairing hippocampal CA1 neurons and improving cognitive function in cardiac arrest patients.

## Data Availability Statement

The raw data supporting the conclusions of this article will be made available by the authors, without undue reservation, to any qualified researcher.

## Ethics Statement

The animal study was reviewed and approved by Institutional Animal Care and Xuzhou Medical University.

## Author Contributions

SH designed the research. SG wrote the main manuscript text. RW and SH helped to polish the manuscript. SG, LS, and JH performed the experiments. SG and RW analyzed data and prepared figures. DH and XZ helped to perform the experiments. YZ participated in the manuscript modification. All authors reviewed the manuscript.

## Conflict of Interest

The authors declare that the research was conducted in the absence of any commercial or financial relationships that could be construed as a potential conflict of interest.

## Publisher’s Note

All claims expressed in this article are solely those of the authors and do not necessarily represent those of their affiliated organizations, or those of the publisher, the editors and the reviewers. Any product that may be evaluated in this article, or claim that may be made by its manufacturer, is not guaranteed or endorsed by the publisher.
